# Investigation of the *RFC1* Repeat Expansion in a Canadian and a Brazilian Ataxia Cohort: Identification of Novel Conformations

**DOI:** 10.3389/fgene.2019.01219

**Published:** 2019-11-22

**Authors:** Fulya Akçimen, Jay P. Ross, Cynthia V. Bourassa, Calwing Liao, Daniel Rochefort, Maria Thereza Drumond Gama, Marie-Josée Dicaire, Orlando G. Barsottini, Bernard Brais, José Luiz Pedroso, Patrick A. Dion, Guy A. Rouleau

**Affiliations:** ^1^Department of Human Genetics, McGill University, Montréal, QC, Canada; ^2^Montreal Neurological Institute and Hospital, McGill University, Montréal, QC, Canada; ^3^Department of Neurology and Neurosurgery, McGill University, Montréal, QC, Canada; ^4^Division of General Neurology and Ataxia Unit, Department of Neurology, Federal University of São Paulo (UNIFESP), São Paulo, Brazil

**Keywords:** ataxia, RFC1, repeat-primed polymerase chain reaction, repeat expansion disease, canvas

## Abstract

A biallelic pentanucleotide expansion in the *RFC1* gene has been reported to be a common cause of late-onset ataxia. In the general population, four different repeat conformations are observed: wild type sequence AAAAG (11 repeats) and longer expansions of either AAAAG, AAAGG or AAGGG sequences. However only the biallelic AAGGG expansions were reported to cause late-onset ataxia. In this study, we aimed to assess the prevalence and nature of *RFC1* repeat expansions in three cohorts of adult-onset ataxia cases: Brazilian (n = 23) and Canadian (n = 26) cases that are negative for the presence of variants in other known ataxia-associated genes, as well as a cohort of randomly selected Canadian cases (n = 128) without regard to a genetic diagnosis. We identified the biallelic AAGGG expansion in only one Brazilian family which presented two affected siblings, and in one Canadian case. We also observed two new repeat conformations, AAGAG and AGAGG, which suggests the pentanucleotide expansion sequence has a dynamic nature. To assess the frequency of these new repeat conformations in the general population, we screened 163 healthy individuals and observed the AAGAG expansion to be more frequent in cases than in control individuals. While additional studies will be necessary to asses the pathogenic impact of biallelic genotypes that include the novel expanded conformations, their occurrence should nonetheless be examined in future studies.

## Introduction

Autosomal recessive cerebellar ataxias regroup a number of heterogenous neurodegenerative diseases. While each form of ataxia exhibits the key feature of cerebellar dysfunction, typically accompanied by gait and balance problems, some forms have distinct clinical characteristics such as, dysarthria, dysmetria or oculomotor abnormalities. Other neurological dysfunctions and/or non-neurologic phenotypes have also been reported in some cases. Among the different forms of recessive ataxia, Friedreich’s ataxia (FRDA) has the highest prevalence and is the most studied (Synofzik et al., 2019). In regard to prevalence FRDA is followed by autosomal recessive spastic ataxia of Charlevoix-Saguenay, ataxia with vitamin E deficiency, autosomal recessive cerebellar ataxia types 1 and 2, and ataxia with oculomotor apraxia types 1 and 2 (Noreau et al., 2013).

Cortese and colleagues established the biallelic expansion of an AAGGG pentanucleotide repeat located in the second intron of the *RFC1* gene (hg19/GRCh37, chr4:39,350,045-39,350,103) to be a frequent cause of late-onset recessive ataxia; this particular expansion was reported to explain over 20% of sporadic ataxia in a cohort of Caucasian cases (Cortese et al., 2019). In the same study, a total of four distinct intronic repeat conformations were also identified: AAAAG_11_, the wild-type sequence, and longer expansions of AAAAG_n_, AAAGG_n_, and AAGGG_n_. The configuration with the AAGGG pentanucleotide was shown to be the only disease-causing conformation of the expansion, ranging in size from 600 to 2,000 repeats.

Considering that *RFC1* appears to be a novel genetic risk factor that explains a significant share of adult-onset ataxia cases, the identification of carriers in other populations may altogether expand its clinical spectrum, provide examples of variable regional prevalence, and uncover repeat sequence differences. Therefore, we screened the *RFC1* expansion in Canadian and Brazilian ataxia patients.

## Materials and Methods

Two cohorts consisting of unrelated adult-onset ataxia cases were used to estimate the prevalence of the *RFC1* expansions. Detailed cohort demographics are shown in **Table 1**. Cohort 1 and cohort 2 comprised Brazilian (n = 23) and Canadian (n = 26) adult-onset cases, who did not carry variants in genes associated with common dominant and recessive ataxias (FRDA, dentatorubral–pallidoluysian atrophy, spinocerebellar ataxia type 1 (SCA1), SCA2, SCA3, SCA6, SCA7, SCA10, SCA12, SCA17, and autosomal recessive cerebellar ataxia type 1). Cohort 3 consisted of randomly selected adult-onset ataxia Canadian probands (n = 128). In addition, a cohort of 163 healthy Canadian control individuals was also examined, to estimate the frequency of the novel sequence conformations that were observed for the *RFC1* repeat expansion. All subjects provided informed consent, and the study was approved by the appropriate institutional review boards.

**Table 1:** Allele frequency of *RFC1* repeat expansions in Brazilian and Canadian ataxia cohorts.

	Cohort 1 n = 23	Cohort 2 n = 26	Cohort 3 n = 128	Control group n = 163
Mean age at onset	37 ± 7	57 ± 10	50 ± 13	
Geographical origin	Brazil	Canada	Canada	Canada
Male:female	13/10	9/17	NA	1
Family history (familial/sporadic)	familial	both	both	–
Prior genetic testing for other common ataxias	+	+	–	–
AAAAG_11_	29 (63%)	36 (69.2%)	193 (75.4%)	276 (84.6%)
AAAAG_n_	6 (13%)	1 (1.9%)	20 (7.8%)	22 (6.7%) 1 homozygous, 20 heterozygous)
AAAGG_n_	3 (6.5%)	3 (5.8%)	8 (3.1%)	8 (2.5%) (8 heterozygous)
AAGGG_n_	5 (10.9%, 1 biallelic, 3 heterozygous)	11 (21.1%, 11 heterozygous)	16 (6.2%) (1 biallelic, 14 heterozygous)	13 (4%) (13 heterozygous)
AAGAG_n_	2 (4.3%)	1 (1.9%)	18 (7%)	7 (2.1%)
AGAGG_n_	1 (2.2%)	0	1 (0.4%)	0
Compound heterozygotes	AAAAG_n_/AGAGG_n_ (1)	0	AAGAG_n_/AGAGG_n_ (1), AAAAG_n_/AAAGG_n_ (1), AAAGG_n_/AAGGG_n_ (1)	AAAAG_n_/AGAAG_n_ (2), AAAGG_n_/AAGGG_n_ (1)

Screening of the *RFC1* repeat expansion was performed on genomic DNA by repeat-primed polymerase chain reaction (RP-PCR) as described in Cortese et al. using the same set of primers (Cortese et al., 2019). RP-PCR products were separated on an ABI3730xl DNA Analyzer (Applied Biosystems^®^, McGill University and Genome Québec Innovation Centre) and results were visualized using GeneMapper^®^ v.4.0 (Applied Biosystems^®^). The samples that seemed biallelic for the AAGGG repeat (according to the RP-PCR results) were subjected to long-range PCR [using the same primers as Cortese et al. (2019)] and Sanger sequencing. Samples for which the allelic repeat combinations could not be determined by RP-PCR were subjected as well to a long-range PCR; the product of which was purified (QIAquick gel extraction kit, Qiagen).

The Sanger sequencing results of these long-range PCR were analyzed using Unipro UGENE version 1.31 (Okonechnikov et al., 2012). Finally, to compare the distribution of *RFC1* alleles in Canadian case and control groups, we performed a Chi-square test using the counts of five conformations (AAAAG_11_, AAAAG_n_, AAAGG_n_, AAGGG_n_, AAGAG_n_) in a 2 × 5 contingency table (**Supplementary Table 2**).

## Results

To examine the prevalence of *RFC1*-based adult-onset ataxia, we screened the nature and size of the repeat expansions in a cohort of Brazilian cases and two cohorts of Canadian cases. The RP-PCR examination of the Brazilian cohort revealed two out of 23 individuals to be carrier of biallelic AAGGG causative expansions. However, long-range PCR and Sanger sequencing subsequently revealed one of these two individuals to actually carry a biallelic AAAGG expansion; the same biallelic expansion was observed in his sister. It therefore appears that expanded AAAGG repeat expansion can sometimes mimic the AAGGG expansion when an assessment is made only by RP-PCR, under such a context the results can lead to a misinterpretation of the true nature of the repeat expansion. The use of different RP-PCR primers (Cortese et al., 2019) could not resolve this mimicry of the AAGGG repeat by the AAAGG repeat.

**Figure 1:**
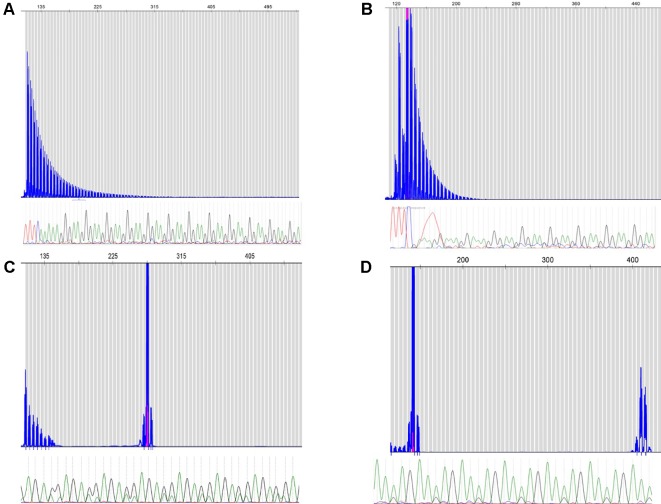
Repeat-primed PCR reactions targeting the AAGGG repeated conformation. Fragment plots and Sanger chromatograms of long-range PCR results are shown for biallelic AAGGG Canadian **(A)** and Brazilian **(B)** patients. Novel AGAGG **(C)** and AAGAG **(D)** expansion conformations (heterozygous) required both methods for identification.

Across the different cohorts, two cases were observed and validated to carry the causative biallelic AAGGG repeat expansion originally reported by Cortese et al. (Cortese et al., 2019). One case with two patients were Brazilian siblings, and the other one was Canadian (**Figures 1A, B** respectively). Clinical features of these three patients with biallelic AAGGG expansions are summarized in **Supplementary Table 1**. The allele count and frequency of the different repeat expansions observed in all three cohorts are shown in **Table 1**.

Interestingly, our cohorts of cases revealed the presence of two previously undescribed repeat expansion confirmations (AAGAG and AGAGG); both motifs were observed by long-range RP-PCR and validated by Sanger sequencing. The RP-PCR plots were characterized by a single peak, but the allele was longer than the wild type (**Figures 1C, D**). The novel conformations were in a heterozygous state in all 22 carrier individuals (**Table 1**). The average length of these novel expand configurations is 800 bp (160 repeats) ranging from 600 to 900. The approximate lengths of the repeat conformations were shown in **Supplementary Figure 1**.

The frequency of the expanded AAGAG and AGAGG repeat configurations was assessed in 163 Canadian control individuals showing no signs of ataxia; using a combined RP-PCR and long-range PCR approach. On the whole, a total of seven control individuals presented a heterozygous AAGAG expanded configurations. None of the control individuals tested presented an expanded AGAGG conformations. The frequency of the novel AAGAG expansion was found to be higher in cases than in controls (7.0% in cases and 2.1% in controls). The allele counts and Chi-square calculation values were shown in **Supplementary Table 2**. The distribution of the different conformations was found to be different in cases and controls (P = 0.022).

## Discussion

This study represents a follow-up examination of the *RFC1* pentanucleotide repeat expansion recently found to cause adult-onset ataxia (Cortese et al., 2019). A total of 49 cases (26 Canadian and 23 Brazilian) for which genetic testing did not reveal the cause of the disease to be a previously identified ataxia gene and an unrelated cohort of 128 adult-onset Canadian cases for who no prior genetic test results was available. The nature and size of the conformation reported to be expanded in *RFC1* was examined using a RP-PCR and long-range PCR sequencing approach. Conversely to what was previously observed in the original study (Cortese et al., 2019), the AAGGG expansion explained a much smaller share of the Brazilian and Canadian cases examined here (0.6% and 4.3% of unrelated cases in Canadian and Brazilian cohorts, respectively by comparison to 22% sporadic Caucasian cases in the study by Cortese and colleagues) (Cortese et al., 2019).

The frequency of the four allelic repeat configurations has been described before, but only in control individuals with no history or signs of ataxia (Cortese et al., 2019). However, the *RFC1* repeat locus could not be assessed in 3% of this earlier examination in control individuals, an observation which led the authors to suggest the existence of additional allelic configurations.

Unlike the previous examinations of the pentanucleotide repeats of *RFC1* in the context of adult-onset ataxia (Cortese et al., 2019; Rafehi et al., 2019), we actually report the observation of two novel repeat conformations (AAGAG and AGAGG) in a heterozygous state. The frequency of the AAGAG repeat was observed to be 0.07 and 0.02 in Canadian cases and controls respectively. The observation of two previously unreported pentanucleotide repeats might represents clues which will lead to a better understanding of the expansion mechanism leading to the pathogenic repeat of *RFC1*. While it is not a conclusive observation, the higher frequency of the novel AAGAG repeat in cases (by comparison to its frequency in control individuals) suggests that it could eventually be observed to also be associated with adult-onset ataxia. Hence additional work might be needed to determine the frequency of other pentanucleotide repeat conformations, and their association to adult-onset ataxia.

Given the dynamic nature of the *RFC1* repeat, multiple validations of sequences and repeat length should be performed. To prevent false positive results, the RP-PCR plots should be interpreted with caution, and each AAGGG-positive sample should be validated by Sanger sequencing to confirm its true sequence.

The presence of disease-associated or wild type repeat sequences has been observed across several expansion-associated diseases, such as SCA37 (Seixas et al., 2017; Loureiro et al., 2019), SCA10 (Matsuura et al., 2006), and FRDA (Al-Mahdawi et al., 2018). Variations interrupting the pure repeat sequences of disease-causing alleles can affect their penetrance, as well as the age at onset and severity of the conditions associated with specific repeats (Al-Mahdawi et al., 2018) Also, interruptions in the normal alleles prevent the disease-associated expansions and provide the stability of repeats in disease-causing alleles. We did not observe the new sequence conformations along with an AAGGG expansion in any of the patients, therefore further studies will be required to determine whether they affect the function or the disease severity.

The low prevalence of the *RFC1* AAGGG expansion, as well as the identification of novel conformations might be due to the different genetic backgrounds of the Canadian and Brazilian populations (Dupré et al., 2006). Although we screened a control group of Canadian individuals to assess the frequency of each repeat conformation in the general population, a limitation of the current study is that the study cohort do not contain a Brazilian control group. Therefore, additional case and control cohorts should be tested for the same repeat, in order to draw a clear conclusion on its frequency in adult-onset ataxia. Further studies are warranted to confirm the structure and sequence of the repeated region, and to investigate potential biological impacts.

## Data Availability Statement

The datasets generated for this study can be found in the GenBank, accession MN630691, MN630692, MN630693, MN630694, MN630695.

## Ethics Statement

The studies involving human participants were reviewed and approved by Research Ethic Board of the Montreal Neurological Institute and Hospital (Neuro), affiliated to the MUHC Research Ethics Board. The patients/participants provided their written informed consent to participate in this study.

## Author Contributions

FA: design and conceptualized the study; analysis and interpretation of the data; and drafting the manuscript for intellectual content. JR: revising the manuscript for intellectual content. CB: analysis and interpretation of the data; and revising the manuscript for intellectual content. CL: revising the manuscript for intellectual content. DR: analysis and interpretation of the data. MG: the acquisition of data. M-JD: the acquisition of data. OB: the acquisition of data, revising the manuscript for intellectual content. BB: the acquisition of data, revising the manuscript for intellectual content. JP: the acquisition of data, revising the manuscript for intellectual content. PD: design and conceptualized the study; interpretation of the data; and drafting or revising the manuscript for intellectual content. GR: design and conceptualized the study; interpretation of the data; and drafting or revising the manuscript for intellectual content.

## Funding

F.A. and C.L. were funded by the Fonds de Recherche du Québec–Santé. J.P.R. has received a Canadian Institutes of Health Research Frederick Banting & Charles Best Canada Graduate Scholarship (FRN 159279).

## Conflict of Interest

The authors declare that the research was conducted in the absence of any commercial or financial relationships that could be construed as a potential conflict of interest.
