# Supplementary material for: Investigation of the RFC1 Repeat Expansion in a Canadian and a Brazilian Ataxia Cohort: Identification of Novel Conformations
Source: Front Genet. 2019 Nov 22;10:1219. doi: 10.3389/fgene.2019.01219 (PMC6884024; doi:10.3389/fgene.2019.01219)
Supplement: Supplementary file 1 [file DataSheet_1.docx]

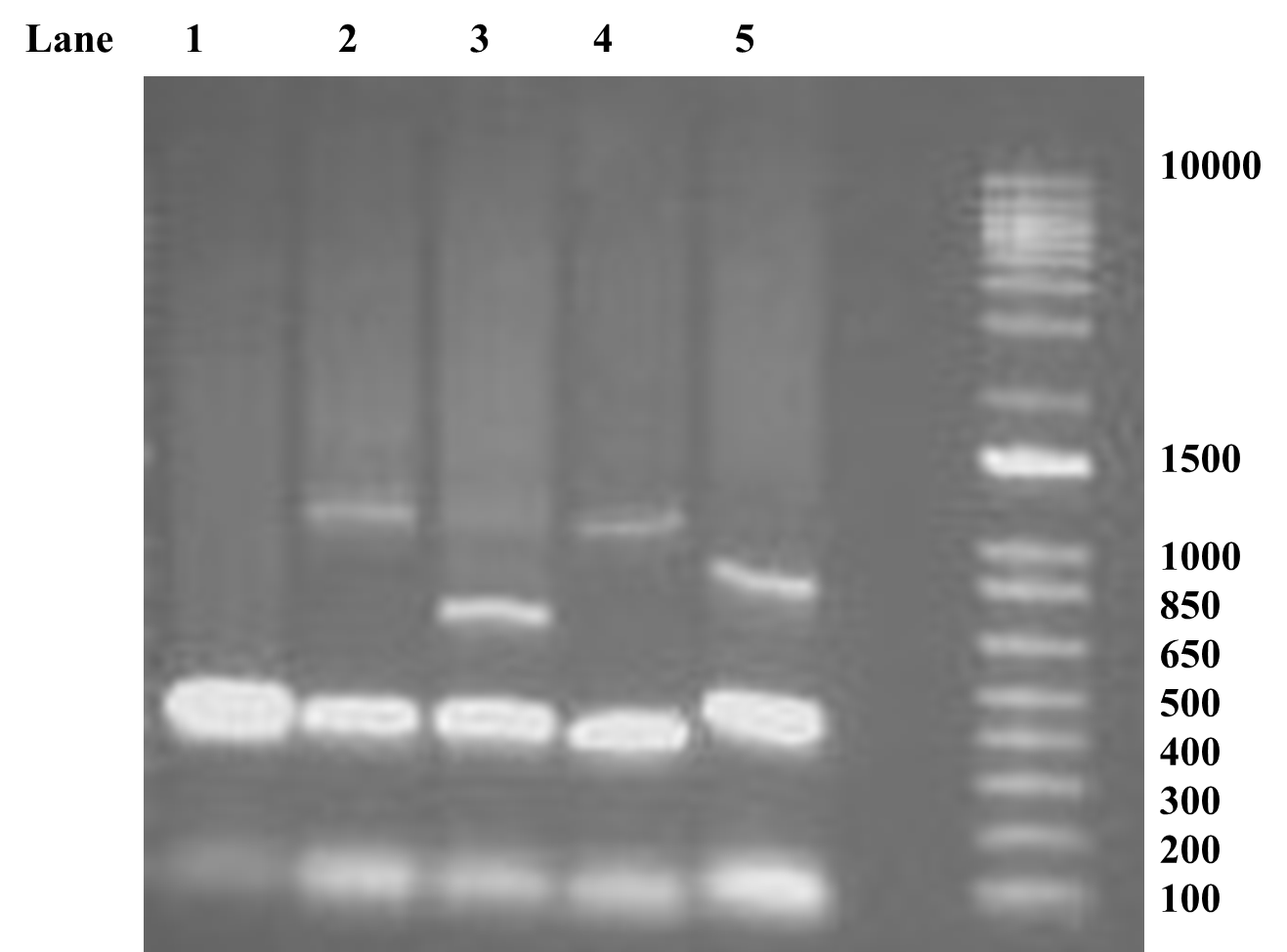


**Supplementary Figure 1**. Long-range PCR amplification using Canadian control samples. Lane 1: wild type AAAAG_11_, lane 2: heterozygous AAAAG_11_ and AAAAG_n_, lane 3: heterozygous AAAAG_11_ (lower band) and AGAAG_n_ (upper band), lane 4: heterozygous AAAAG_11_ (lower band) and AAAGG_n_ (upper band), lane 5: heterozygous AAAAG_11_ (lower band) and AAGGG_n_ (upper band).

**Supplementary Table 1.** Clinical features of patients carrying the recessive AAGGG repeat expansion in *RFC1*

| sample | origin | gender | family history | age at onset | age at examination | symptom at onset | neuropathy | cerebellar ataxia | nystagmus | cerebellar atrophy | SARA | other |
| --- | --- | --- | --- | --- | --- | --- | --- | --- | --- | --- | --- | --- |
| Fam I-I | Brazilian | female | yes (affected sister, unaffected parents) | 45 | 58 | Dizziness, gait and balance problems | sensorimotor axonal polyneuropathy | yes | yes | yes | 25 | Dysarthria, brisk tendon reflexes, vestibular areflexia |
| Fam I-II | Brazilian | female | yes (affected sister, unaffected parents) | 45 | 56 | Dizziness, gait and balance problems | sensorimotor axonal polyneuropathy | yes | yes | yes | 27 | Dysarthria, brisk tendon reflexes, vestibular areflexia |
| Fam II-I | Italian | female | yes (affected brother) | 55 | 58 | Dizziness, gait and balance problems | none | yes | yes | yes | NA | Abnormal somatosensory evoked potentials, brisk tendon reflexes |

*SARA: Scale for the assessment and rating of ataxia. For vestibular areflexia, a video-head impulse test and caloric test reflex were performed.

**Supplementary Table 2.** The allele counts 2×5 contingency table and Chi- square calculations. χ2  =  11.429, df  =  4, χ2/df  =  2.86, P(χ2 > 11.429)  =  0.0221.

|  | Chi-square calculations for *RFC1* repeat conformations | | | | |
| --- | --- | --- | --- | --- | --- |
|  |  |  |  |  |  |
|  | AAAAG_n_ | AAAAG_n_ | AAGAG_n_ | AAAGG_n_ | AAGGG_n_ |
| Cases  (256) | 193 | 20 | 18 | 8 | 16 |
|  | *205.84* | *18.43* | *10.97* | *7.02* | *12.73* |
|  | (0.8) | (0.13) | (4.5) | (0.14) | (0.84) |
| Controls  (326) | 276 | 22 | 7 | 8 | 13 |
|  | *263.16* | *23.57* | *14.03* | *8.98* | *16.27* |
|  | (0.63) | (0.1) | (3.52) | (0.11) | (0.66) |
|  | 469 | 42 | 25 | 16 | 29 |

*Expected values are displayed in italics. Individual χ2 values are displayed in (parentheses).
